# Metastatic Renal Cell Carcinoma Presenting a Maxillary Mucosal Lesion as a First Visible Sign of Disease: A Case Report and Review of Literature

**DOI:** 10.3390/diagnostics15070938

**Published:** 2025-04-07

**Authors:** Umma Habiba, Abu Faem Mohammad Almas Chowdhury, Rafiz Ahmed, Saiyka S. Chowdhury, Raihanul Ferdoush, Koki Ise, Harun ur Rashid, Zillur Rahman, Zen-ichi Tanei, Shinya Tanaka, Asad-Uz Zaman

**Affiliations:** 1 Department of Oral Pathology & Periodontology, Sapporo Dental College & Hospital, Dhaka 1230, Bangladesh; rafizahmed@gmail.com (R.A.); raihanferdoush@gmail.com (R.F.); azaman@sdch.edu.bd (A.-U.Z.); 2 Department of Cancer Pathology, Faculty of Medicine, Hokkaido University, Sapporo 060-8638, Japan; isek0k126@gmail.com (K.I.); tanei@med.hokudai.ac.jp (Z.-i.T.); tanaka@med.hokudai.ac.jp (S.T.); 3 Department of Conservative Dentistry & Endodontics, Sapporo Dental College & Hospital, Dhaka 1230, Bangladesh; chowdhuryafma@gmail.com; 4 Department of Obstetrics, Gynecology and Reproductive Sciences, School of Medicine, University of Maryland, Baltimore, MD 21201, USA; saiyka.chowdhury@som.umaryland.edu; 5 Department of Oral & Maxillofacial Surgery, Dhaka Dental College & Hospital, Dhaka 1206, Bangladesh; maxfaceharun22@gmail.com; 6 Department of Pathology, Bangabandhu Sheikh Mujib Medical University, Dhaka 1000, Bangladesh; drzillur@bsmmu.edu.bd; 7 Department of Pathology, Hokkaido University Hospital, Sapporo 060-8648, Japan

**Keywords:** oral metastasis, RCC: renal cell carcinoma, renal clear-cell carcinoma (ccRCC), CT: computed tomography, TKI: tyrosine kinase inhibitor, oral mucosa, maxilla

## Abstract

**Background and Clinical Significance:** Renal cell carcinoma (RCC) is the third most common cancer that metastasizes to the oral and maxillofacial region following breast and lung cancers. Metastatic involvement in the oral cavity is rare and can present as a diagnostic challenge due to non-specific clinical features that mimic other benign or malignant conditions. The limited information available regarding oral metastasis of RCC highlights the importance of recognizing this uncommon presentation. **Case Presentation:** A 50-year-old female presented with a painful swelling in the buccal and palatal mucosa of the right maxilla that progressively enlarged over several months. Initially, this lesion was diagnosed clinically as a pyogenic granuloma. However, given the lesion’s continued growth and unusual presentation, a biopsy was performed. Histopathological examination confirmed the lesion as metastatic renal clear-cell carcinoma (ccRCC), with immunohistochemical analysis verifying the renal origin. Further diagnostic tests, including a computed tomography (CT) urogram, chest CT, and bone scintigraphy, revealed additional metastases in the left adrenal gland, lungs, and bone. **Conclusions:** This case is notable because the oral lesion was the first visible sign of RCC, making it a rare presentation of metastatic RCC. This underscores the importance of thorough history taking, detailed clinical evaluations, and considering rare metastatic conditions in the differential diagnosis of oral swellings. Additionally, this case reinforces the significance of routine cancer screenings for early detection of undiagnosed cancer. We also updated a previous literature review of metastatic RCC to the head and neck region, covering cases until 2023.

## 1. Introduction

The oral cavity is generally an infrequent site of cancer metastasis, accounting for approximately 1% of all cases of neoplasm in the head and neck region [1,2,3,4]. The most common primary tumors that metastasize to the oral cavity are lung carcinoma in males and breast carcinoma in females. Other primary tumors that can spread to the oral cavity include renal cell carcinoma (RCC), liver cancer, and colorectal cancer, although they are less frequent [5,6,7]. Oral metastases are common in the gingiva, tongue, and jawbone, though other locations within the mouth may also be affected [8].

RCC, which accounts for about 2.2% of malignant neoplasms in adults, has a high potential for metastasis. It is estimated that roughly one-third of individuals diagnosed with RCC will develop metastases, and of those, half will have distant metastases from the original tumor site. The most common sites of distant metastasis of RCC include the lungs, regional lymph nodes, abdominal lymph nodes, bones, contralateral kidney, adrenal glands, brain, and liver [6].

Metastases of RCC to the head and neck region are relatively rare. However, when it occurs, it is most commonly found in areas such as skin, subcutaneous lymph nodes, nasal cavities, paranasal sinuses, parotid gland, tongue, oral mucosa, tonsils, orbit, mandible, and maxilla [9]. The clinical presentation of RCC metastasis in the mouth can be diverse based on the specific anatomical site involved [10]. These metastases may be presented as painful or asymptomatic, rapidly growing nodules. They can be accompanied by metastatic cervical adenopathy, epistaxis, anosmia, facial pain, nasal obstruction, diplopia, or paresthesia [11,12,13,14]. These diverse symptoms highlight the complexity of RCC metastasis in the head and neck, often mistaken for other, more common conditions, leading to delayed diagnosis.

In infrequent instances, oral metastasis may be the first presenting sign of RCC. When it is the first manifestation, it generally suggests an advanced stage of disease and is often associated with a poorer prognosis. This is because by the time the cancer reaches the oral cavity, it has likely already spread to other distant sites, and treatment options may be more limited. Early detection of oral metastasis is crucial for improving patient outcomes, as prompt and aggressive treatments like surgery or targeted therapies may help extend survival and improve quality of life [15]. However, because oral metastases are rare, they are often overlooked in differential diagnoses, leading to delays in treatment. Advances in diagnostic tools and imaging technologies have increased the ability to detect such metastases earlier, contributing to better patient outcomes [16].

In the current report, we present a case of metastatic RCC in the right maxillary mucosa, specifically involving both the palatal and buccal mucosa of a 50-year-old female patient. This oral manifestation was the first and only sign of her underlying renal malignancy. Subsequent investigations revealed additional distant metastasis in her left adrenal gland, lung, and bones, underscoring the aggressive and widespread nature of the disease once oral symptoms appear. This case is unique because it involves an atypical site for metastasis and was the initial manifestation of an otherwise unidentified primary malignancy. In addition, we performed a literature review of studies published between December 2023 and January 2025, focusing on case reports of renal clear-cell carcinoma (ccRCC) metastasis to the head and neck to provide an updated understanding of these metastases.

## 2. Detailed Case Description

A 50-year-old female patient reported to the Oral and Maxillofacial Surgery Department at Dhaka Dental College and Hospital in Bangladesh, complaining of painful swelling on the right maxilla. The swelling has been progressively expanding over the past few months. The patient reported that the mass was impairing her ability to eat and had caused several instances of mild oral bleeding. During her hospitalization, she also developed multiple episodes of epistaxis, which further indicated the extent of her systemic involvement. To evaluate her overall health, a comprehensive blood test was performed, revealing that she was severely anemic, with a hemoglobin level of only 5.5 g/dL. To address the anemia, the patient was transfused with two units of whole blood and one unit of red cell concentrate.

The patient’s past medical history revealed additional concerns, including episodes of hip and back pain and hematuria. These symptoms raised concerns about a potential underlying systemic condition that could account for the maxillary swelling and other systemic signs, including anemia and epistaxis. As a result, a more comprehensive diagnostic assessment was initiated.

**Figure 1:**
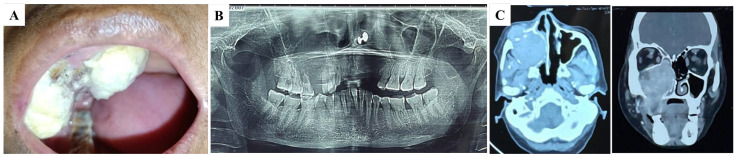
Clinical, radiographical, and CT scans of the oral and maxillofacial region. (**A**) Intra-oral view of the initial lesion. A double lobe nodule is observed in the right maxilla. (**B**) A panoramic radiograph displays a radiolucent lesion with an indistinct margin, suggesting osteolysis between teeth 12 and 17. (**C**) A CT scan of the maxillofacial area shows a soft tissue lesion in the right maxillary sinus with heterogeneous enhancement and destruction of the bony wall.

Upon performing an intraoral examination, a double-lobed nodule involving both the buccal and palatal mucosa was identified in the right maxilla (Figure 1A). The lesion measured approximately 60 mm × 50 mm × 30 mm and was covered with a white pseudomembranous surface. There were no signs of regional lymphadenopathy, and a complete head and neck examination revealed no other abnormalities. An incisional biopsy was performed to obtain tissue for histopathological analysis. The biopsy results indicated that the lesion was highly suggestive of renal clear cell carcinoma (RCC).

Radiographic imaging was subsequently conducted to evaluate the extent of the lesion and its relationship with the surrounding structures. A panoramic radiograph revealed a radiolucent lesion with a diffuse border extending from the distal aspect of the right lateral incisor (#12) to the mesial surface of the right second molar (#17). This indicated osteolysis between teeth 12 and 17 (Figure 1B). To gain more detailed insight into the lesion and its behavior, a CT scan of the maxillofacial region was performed. The scan revealed a moderate, heterogeneous enhancing soft tissue density lesion in the right maxillary sinus with destruction of the surrounding bony wall. The lesion extended into the oral cavity, involving the right buccal mucosa and the roof of the mouth. Additionally, mucosal thickening was observed in the right frontal, sphenoid, and ethmoid sinuses, suggesting that the tumor had spread beyond the maxillary sinus and affected nearby sinuses (Figure 1C).

**Figure 2:**
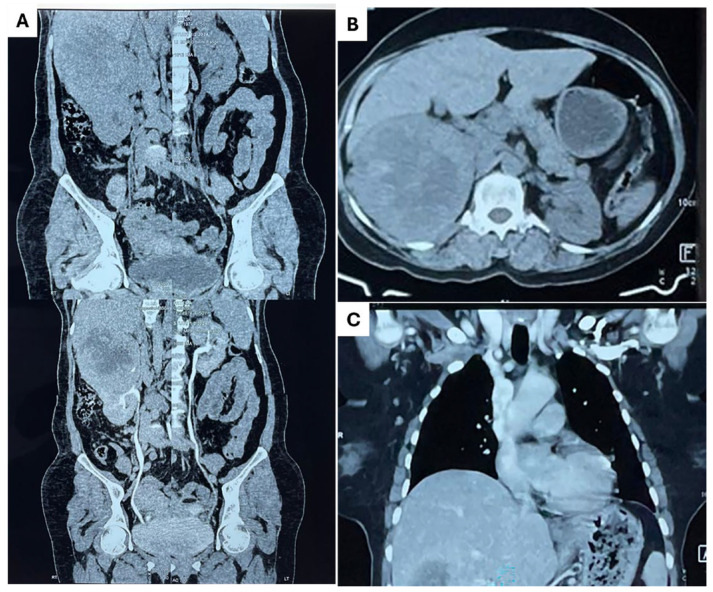
CT urogram and CT scan of the lung. (**A**,**B**) The CT urogram of the kidney showed a large, heterogeneous, calcified mass with mixed density and enhancement in the upper pole of the right kidney, along with an additional mass in the left adrenal area. (**C**) The chest CT scan revealed metastatic lesions in the lungs.

**Figure 3:**
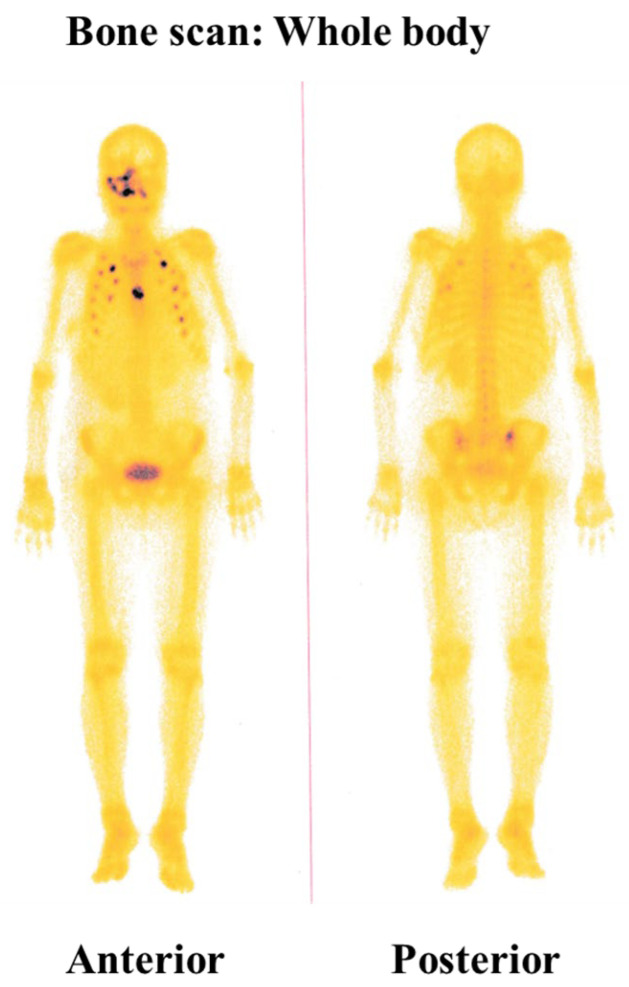
Bone scintigraphy. Bone scintigraphy shows intense radiotracer concentration in the maxillofacial area. Additionally, multiple focal areas of increased radiotracer concentration are seen in several ribs and the sternum.

A CT urogram was conducted to investigate the patient’s overall condition further and evaluate potential metastatic involvement. The scan revealed a large, heterogeneous, mixed-density, calcified, enhancing mass lesion with central necrosis in the upper polar region of the right kidney posteriorly. The mass measured approximately 9.8 cm × 9.8 cm × 11.0 cm. Another heterogenous enhancing mass lesion was noted in the left adrenal region, measuring 4 cm × 4.2 cm × 6.6 cm. The size and characteristics of the right renal mass suggest it was the origin of the primary tumor (ccRCC), while the left adrenal mass appeared to be a secondary metastasis (Figure 2A,B). A chest CT scan was also performed, which revealed multiple pulmonary nodules in both lungs and mediastinal lymphadenopathy, suggesting secondary deposits (Figure 2C). Additionally, bone scintigraphy revealed multiple focal areas of increased radiotracer uptake in multiple ribs on both sides and the sternum, indicating that the disease had metastasized to the bones (Figure 3). These findings confirmed that the patient’s disease had spread systemically.

**Figure 4:**
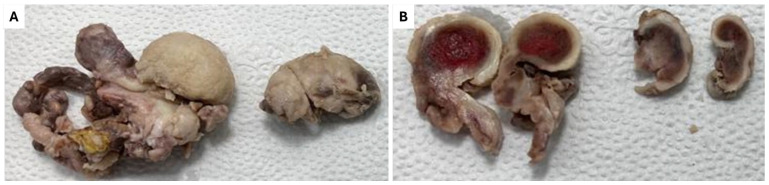
Gross specimen following surgical removal. (**A**) Operation materials are received as mucosa-covered white pieces of tissue. (**B**) The cut surfaces reveal blood-filled central areas.

**Figure 5:**
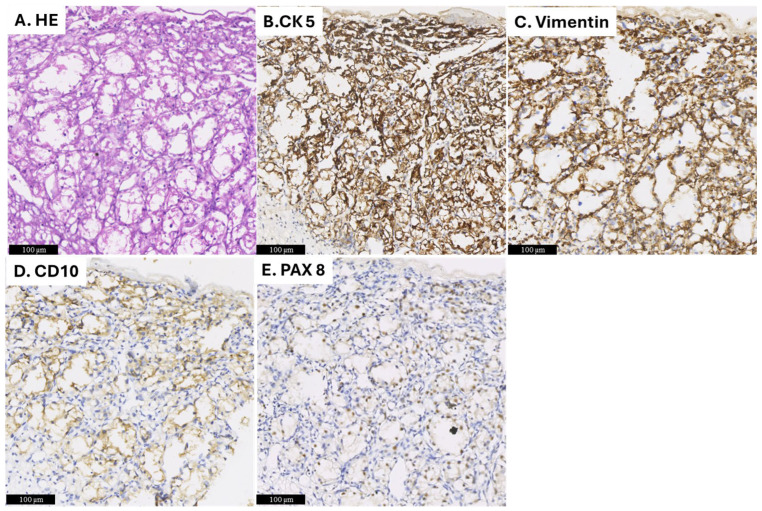
Histopathological findings. (**A**) HE staining reveals large nests of tumor cells characterized by round to polygonal-shaped cells with abundant clear cytoplasm and centrally located nuclei. (**B**–**E**) The cancer cells are positive for CK5, Vimentin, CD10, and PAX8.

Given the systemic spread of the disease, the patient was scheduled for surgery to remove the maxillary lesion. Following all aseptic precautions, a Weber–Fergusson incision was made on the right side of the face, and the lesion was excised. An antibiotic-soaked gauze pack was placed in the surgical site to prevent infection and promote healing. The excised tissue was sent for histopathological examination, which consisted of two pieces of tissue, partly covered by mucosa, with dimensions of 6 × 5 × 3 cm and 4 × 2.5 × 2.5 cm. The cut surface of the tissue appeared grey and brown and contained blood-filled central areas (Figure 4A,B). Microscopic examination showed an ulcer and necrotic tumor cells. The viable tumor showed large nests of tumor cells comprised of round to polygonal cells with abundant clear cytoplasm and centrally placed nuclei. Delicate septa with thin-walled blood vessels were seen separating the tumor nests. (Figure 5A). Immunohistochemical staining was performed, revealing that the tumor cells were positive for CK5, Vimentin, CD10, and PAX8, which supported the diagnosis of ccRCC (Figure 5B–E).

Based on the histopathologic findings and immunohistochemical results, a diagnosis of ccRCC was confirmed. Systemic evaluations revealed metastases in the left adrenal gland, lungs, and bones, indicating that the patient had advanced-stage disease. The patient was advised to undergo a CT-guided core biopsy of both the right renal mass and lung nodule to evaluate the extent of metastatic spread further. However, due to financial constraints, the patient decided not to proceed with further diagnostic tests or additional surgical procedures.

When diagnosed, the patient was prescribed Pazopanib, a multi-tyrosine kinase inhibitor (TKI) that targets multiple receptors, including vascular endothelial growth factor receptor (VEGFR) 1 to 3, platelet-derived growth factor receptor (PDGFR)-α and -β, fibroblast growth factor receptor (FGFR)-1 and -3, cytokine receptor (Kit), interleukin-2 receptor-inducible T-cell kinase (Itk), lymphocyte-specific protein tyrosine kinase (Lck), and the transmembrane glycoprotein receptor tyrosine kinase (c-Fms). Pazopanib 800 mg was prescribed as an oral tablet to be taken daily. Additionally, an anti-ulcer medication was also prescribed to manage the potential side effects of the treatment.

**Figure 6:**
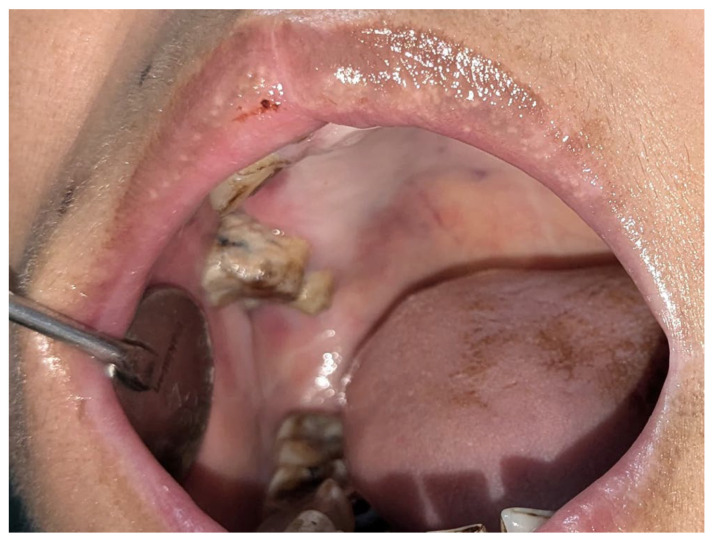
Follow-up clinical photograph.

Six months later, a follow-up showed that the removed oral lesion had healed entirely without recurrence (Figure 6). However, she reported generalized weaknesses, difficulty eating, and episodes of epistaxis.

Complete regression of the intraoral lesion is seen 6 months after the surgery.

## 3. Discussion

### 3.1. Overview of Renal Cell Carcinoma Metastasis

Renal cell carcinoma (RCC) is an aggressive malignancy that originates in the kidneys, and it is known for its potential to metastasize to various distant organs. Of the cancers that typically metastasize to the head and neck area, RCC ranks as the third most common malignancy, with lung and breast cancers being other frequent sources of metastasis [5]. RCC poses a significant risk to patients, especially since it can silently metastasize in its early stages, often without noticeable symptoms until the disease progresses. Notably, approximately 20% of oral metastasis cases are identified before the primary tumor, underscoring the challenges of diagnosing RCC in its early stage [1,2,7,8,16].

The metastatic spread of RCC to the oral cavity typically occurs through both arterial and venous routes. The venous system, particularly Batson’s plexus, is the key pathway for disseminating RCC to distant organs. Batson’s plexus is a venous system that bypasses the pulmonary filtration system, allowing cancerous cells to spread directly from the kidneys to other regions without first passing through the lungs. As a result, RCC can often metastasize to multiple distant sites, and approximately two-thirds of patients with RCC experience widespread metastasis, contributing to the disease’s complexity and severity [17].

According to the literature reviews, oral metastasis from RCC most often occurs in older individuals, typically those over the age of 60. Metastases are more prevalent in men, reflecting the higher incidence of RCC metastasis in males. The most common oral sites affected by RCC metastases are the posterior mandible, gingiva, and tongue, which are highly vascularized regions. However, oral metastasis to the buccal and palatal mucosa is rare, considering the area a rare metastatic site. The most common clinical manifestation is a mass or nodule, while pain and bleeding are the most frequent symptoms [12,18,19,20,21,22]. The present case illustrates an atypical presentation of RCC metastasis in the buccal and palatal mucosa of the maxilla, a location that has not been widely documented in the literature. This emphasizes the importance of clinicians staying alert to the oral manifestation of RCC, even in atypical locations.

### 3.2. Diagnostic Challenges and Clinical Evaluation

The diagnosis of RCC is often delayed because RCC typically lacks symptoms in its early stages, especially in patients with no history of malignant diseases. Oral metastasis can be the first clinical manifestation of a primary tumor, as reported in reviews by Vanja et al. [18] and the current study, occurring in 36.4% and 33% of cases, respectively. Although oral metastasis is commonly associated with advanced disease, where patients often have metastases in other organs, it can, in rare instances, be the initial sign of RCC spread [10,23,24]. A previous study reported that approximately 25% of patients present with oral lesions before other distant metastases are detected [15]. In the present case, the oral lesion was the first manifestation of RCC, ultimately leading to the identification of multiple distant metastases, highlighting the importance of considering metastatic RCC in patients with unexplained oral lesions.

When oral RCC metastasis does occur, it may present with aggressive clinical features, such as rapidly growing exophytic masses, spontaneous bleeding, and occasionally asymptomatic submucosal nodules [7]. The challenge in diagnosing such lesions arises from their clinical resemblance to a wide range of other oral conditions, including giant cell granuloma, hemangioma, and pyogenic granuloma. Furthermore, due to the rich vascularization that characterizes this neoplasm, oral metastatic RCC can show spontaneous or stimulated bleeding, making differentiation even more difficult [14]. In the case described here, the patient experienced repeated episodes of epistaxis linked to the vascular nature of the metastatic RCC.

### 3.3. Pathological Differential Diagnosis and Imaging Techniques

If a patient presents with oral lesions and has no previous history of RCC, the diagnosis of metastasis should be supported by histology. Immunohistochemistry (IHC) is essential in confirming the presence of RCC. The primary markers used to confirm RCC in metastatic lesions are CD10 and PAX8. CD10 is expressed in roughly 98% of RCC cases, while PAX8 shows positivity in approximately 90% of cases. Additional immunohistochemical markers used to diagnose RCC include pan-keratin AE1/AE3, renal cell carcinoma (RCC) antigens, and vimentin [25]. These markers aid in distinguishing RCC from other malignancies with similar histological features, such as salivary gland clear cell carcinomas, which may resemble RCC morphologically but differ in their vascularization pattern and tumor cell arrangement [26].

In addition, recent advancements in imaging technologies, such as Magnetic Resonance Imaging (MRI), CT scans, and bone scintigraphy, have enhanced the ability to detect synchronous metastatic RCC at multiple sites, leading to better disease identification and staging. Bellin et al. recently highlighted significant progress in RCC imaging technologies, such as photon-counting detector CT, dual-energy CT, and high-resolution multiparametric MRI, to continually improve diagnostic accuracy for the early detection of tumors [27].

### 3.4. Treatment Options and Prognosis

Unfortunately, the prognosis for oral metastasis of RCC is poor, with a low survival rate and an average survival time of only a few months after diagnosis. However, treatment strategies can improve outcomes depending on disease staging, the extent of metastasis, and the patient’s overall health. Surgical excision, when feasible, is considered the most effective treatment regardless of the disease stage [28]. RCC is mainly resistant to radiation, and chemotherapy agents like interleukin-2, interferon alpha, and 5-fluorouracil may be beneficial, especially when residual disease remains after surgery. However, the response rate is typically below 25% [4,5,24]. Recent studies suggest combining immunotherapy with anti-VEGFR (vascular endothelial growth factor receptor) tyrosine kinase inhibitors (TKIs) to provide patients with a more targeted and effective treatment option [29]. According to the guidelines from the European Association of Urology (https://uroweb.org/guideline/renal-cell-carcinoma/, accessed on 27 March 2025) and the American Urological Association (https://www.auanet.org/guidelines/renal-cell-carcinoma, accessed on 27 March 2025), cytoreductive nephrectomy is generally recommended for patients with metastatic renal cell carcinoma (mRCC) who have a good performance status, and significant symptoms related to the tumor, such as hematuria or severe distress. However, during diagnosis, the patient did not present symptoms like hematuria or major distress. After assessing the clinical factors, including the patient’s metastatic disease burden, it was concluded that cytoreductive nephrectomy would not provide significant benefits at this stage. Consequently, she was treated with a tyrosine kinase inhibitor (TKI). Due to financial constraints, the patient could not undergo additional investigations to evaluate metastasis in other organs. Therefore, during follow-up, we lack information on the presence or progression of metastasis beyond the oral cavity, lungs, adrenal glands, and bones.

Early detection of RCC is crucial for improving patient survival rates. A recent review by Kase AM et al. [30] shows that patients with regional disease have a 70% five-year survival rate, while those with distant metastases have only a 13% survival rate. This emphasizes the importance of early detection of metastatic lesions, which is difficult without signs or symptoms.

In regions like Bangladesh, where cancer screening programs are not routine and healthcare access is limited, many patients are diagnosed too late for curative interventions. This results in fewer treatment options and poorer prognoses. This case stresses the value of routine cancer screenings for the early detection of any initial signs of undiagnosed cancer.

### 3.5. Review of Literature

A literature search was conducted to examine previous studies on the occurrence of oral metastases from renal cell carcinoma (RCC). A comprehensive review by Vanja et al. [18] analyzed 156 studies documenting 226 cases of RCC metastasis to the head and neck region from 1928 to 2023. Of the 100 studies (64%) included, renal clear cell carcinoma (ccRCC) was identified as the most common histological type, accounting for 122 cases (54%). The review highlighted that soft tissues were more frequently affected by ccRCC metastases than hard tissues. In these 122 cases, 84 were male and 38 were female, with a male-to-female ratio of 2.21:1, and the average age was 60.8 years. The tongue was the most frequently affected site (25.4%), followed by the gingiva (17.2%), parotid gland (13.1%), mandibular bone (12.2%), maxillary bone (11.4%), buccal mucosa and lips (4.9%), hard palate (4%), submandibular gland and soft palate (1.6%), and lymph nodes, tonsils, oral floor, and masticatory space (0.8%). Notably, in 33.6% of cases, the development of oral metastasis was the first clinical indication of a primary renal tumor.

We conducted a literature review of studies published between December 2023 and January 2025 [31,32,33,34,35,36,37,38,39,40,41,42]. The selected time frame was chosen because the most recent comprehensive survey on metastatic RCC to the head and neck regions, by Vanja et al., covered cases from 1928 to 2023. Duplicate cases were excluded from our review. We specifically focused on case reports of ccRCC metastasis to the head and neck to provide an updated understanding of these metastases. Our review identified 12 new cases (excluding the current case) of ccRCC metastasis to the head and neck, with the details summarized in Table 1. This includes the author(s), year of publication, age, gender, whether it was the first sign of metastatic disease, and information on other metastatic sites. The results of our review are as follows:

Similar to the previous review, soft tissues were more commonly affected by ccRCC metastases than hard tissues. Men were predominantly affected, with a male-to-female ratio of 2:1 and an average age of 65.3, slightly older than the average age reported in the previous review. The gingiva was the most frequently affected site (33.3%), followed by the oral mucosa (25%, including buccal, palatal, and alveolar areas), the gingiva (16.6%), and the mandibular bone (8.3%). New sites identified in our review that were not reported in the previous review include the submandibular gland (8.3%) and the maxillary sinus (8.3%). Additionally, in 4 of the 12 cases (33%), oral metastasis was the first indication of an undiagnosed renal tumor discovered during further diagnostic evaluation. Interestingly, all cases had already exhibited metastasis to other sites by the time oral manifestations appeared as the first sign of the disease. These findings align with the previous review.

While many of the findings from both reviews are consistent, some differences were noted in the specific sites of metastasis. These differences in the site may reflect the smaller sample size in our current review (12 cases) compared to the larger dataset in Vanja et al.’s review (122 cases). However, both reviews provide essential insights that enhance our understanding of ccRCC metastasis to the head and neck region.

## 4. Conclusions

Renal cell carcinoma (RCC) is known to metastasize to the head and neck region, though such occurrences are rare. In the present case, the oral lesion was the initial complaint of the disease, prompting further investigation into the primary source and location. A thorough patient history, including past illnesses and presenting symptoms, and a comprehensive physical examination are crucial for understanding the patient’s condition. Furthermore, integrating clinical, radiological, and systemic findings alongside detailed histological analysis is essential for an accurate diagnosis. Since these lesions could be the initial sign of an undiagnosed cancer, routine cancer screenings are necessary for early detection and a more favorable prognosis.

## Figures and Tables

**Table 1:** Data on cases of clear-cell RCC (ccRCC) in the head and neck region.

Authors	Year	Site	Gender	Age	First Sign of Disease	Another Metastasis
Huang and Yu [31]	2023	Gingiva	M	48	No	No metastasis
Justin P. Mehr et al. [32]	2023	Maxillary sinus	F	74	Yes	Lung, adrenal gland
Khushbu et al. [33]	2024	Gingiva	F	52	No	Lung
Bruckmann et al. [34]	2024	Palata mucosa	F	60	No	No metastasis
Camila et al. [35]	2024	Gingiva	M	78	No	No metastasis
Clara et al. [36]	2024	Mandibular bone	M	56	No	No metastasis
Florencia et al. [37]	2024	Maxillary alveolar ridge	F	75	No	Multiple body metastasis
Gabriely et al. [38]	2024	Buccal mucosa	M	55	Yes	Abdomen, bone
Hyeok Tae et al. [39]	2024	Tongue	M	72	No	Lung, bone
Dr. Matías et al. [40]	2024	Submandibular gland	M	73	No	No metastasis
Shuo Liu et al. [41]	2025	Tongue	M	62	Yes	Lung
Yusuke et al. [42]	2025	Gingiva	M	79	Yes	Lung
Habiba et al. (present study)		Buccal and palatal mucosa	F	50	Yes	Lung, adrenal gland, bone

## Data Availability

No new data were created or analyzed in this study. Data sharing is not applicable to this article.
